# An esophageal stent placement in a narrow distal esophagus stricture: A case report

**DOI:** 10.1016/j.radcr.2025.06.091

**Published:** 2025-07-29

**Authors:** A.K. Eppy Buchori, Pramiadi, Aditia Nugraha, Harry Galuh Nugraha, Hilman, Leni Santiana, Dian Komala Dewi

**Affiliations:** a Department of Radiology, Faculty of Medicine Padjadjaran University, Dr. Hasan Sadikin Hospital Bandung, West Java, Indonesia; b Karawang Regional General Hospital, West Java, Indonesia

**Keywords:** Stent, Distal esophagus, Stricture, Fluoroscopy, Case report

## Abstract

An esophageal stricture is defined as an abnormal narrowing of the esophageal lumen. Esophageal stents are used to treat both benign and malignant esophageal strictures. Placement of stents can be particularly challenging in distal and severely narrowed esophageal strictures. In this report, we present the case of an 86-year-old woman with a narrow distal esophageal stricture, successfully treated with the insertion of a self-expanding metal stent (SEMS) with an antireflux feature. The procedure was performed under fluoroscopic guidance. This case aims to provide a valuable reference for medical professionals encountering similar cases requiring esophageal stenting.

## Introduction

Esophageal stricture refers to the abnormal narrowing of the esophageal lumen, often resulting from chronic inflammation, fibrosis, or neoplasia. Strictures are categorized as benign or malignant and commonly present with symptoms such as dysphagia, odynophagia, food impaction, chest pain, and weight loss [1,2].

Over the past decade, esophageal stenting has significantly advanced, particularly with the development of self-expanding plastic stents (SEPS) and self-expanding metal stents (SEMS), which offer a cost-effective and relatively safe treatment for esophageal pathologies [[3], [4], [5]]. Stenting, however, presents challenges—especially in strictures located proximally or distally within the esophagus. Such strictures require careful assessment, as the stent must span the lesion and extend beyond both ends [6].

Distal esophageal strictures are particularly difficult due to the risk of gastroesophageal reflux and aspiration when stents cross the gastroesophageal junction [5]. This report discusses the successful placement of a SEMS with an antireflux valve in an elderly patient with a narrow distal esophageal stricture, performed with fluoroscopic assistance.

## Case report

An 86-year-old woman presented with progressive dysphagia over the past 8 months. Initially limited to solid foods, her symptoms had progressed to include liquids. She described a sensation of food becoming stuck in her lower chest, particularly with rice or meat intake. She also experienced worsening nighttime heartburn and acid regurgitation, a history consistent with long-standing gastroesophageal reflux disease (GERD), previously managed with intermittent use of antacids and proton pump inhibitors (PPIs). She also reported mild postprandial chest pain and an unintentional weight loss of approximately 5 kg over four months. Her past medical history was significant for GERD and hypertension, controlled with amlodipine. She denied vomiting, hematemesis, or melena, and there was no family history of gastrointestinal malignancy. Her diet mainly included traditional Indonesian foods, with a preference for fried and spicy dishes, which she had recently reduced due to her GERD symptoms.

**Fig. 1:**
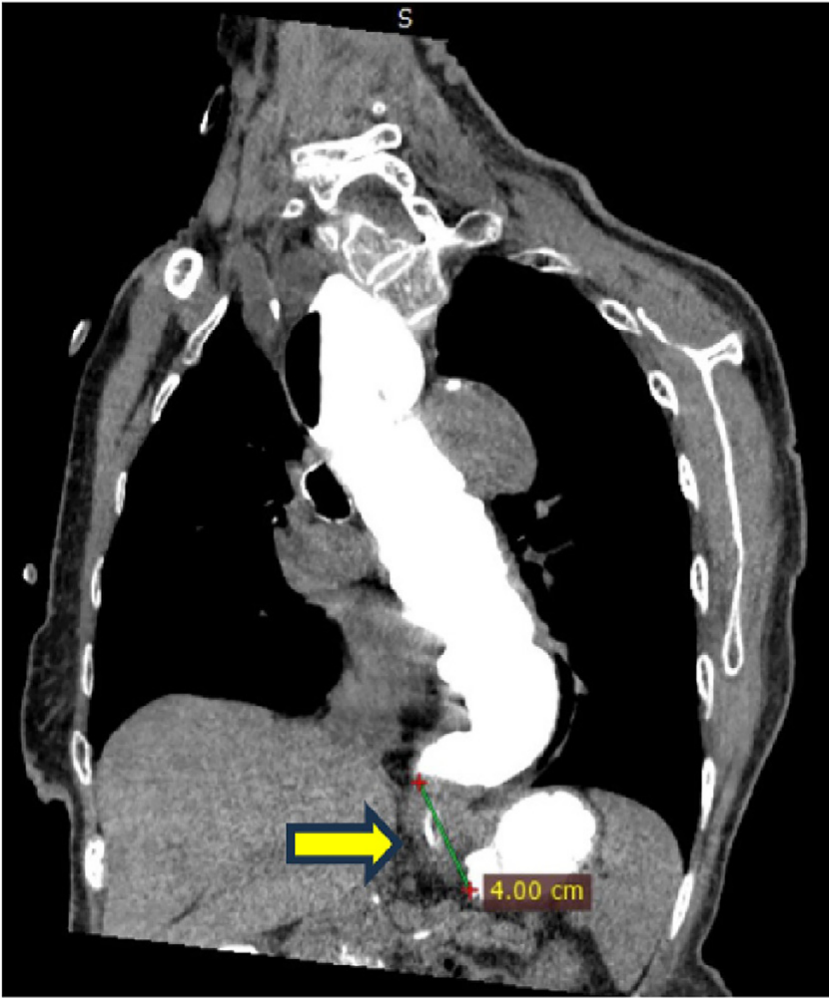
Distal esophageal stricture shown on contrast computed tomography (CT) scan.

**Fig. 2:**
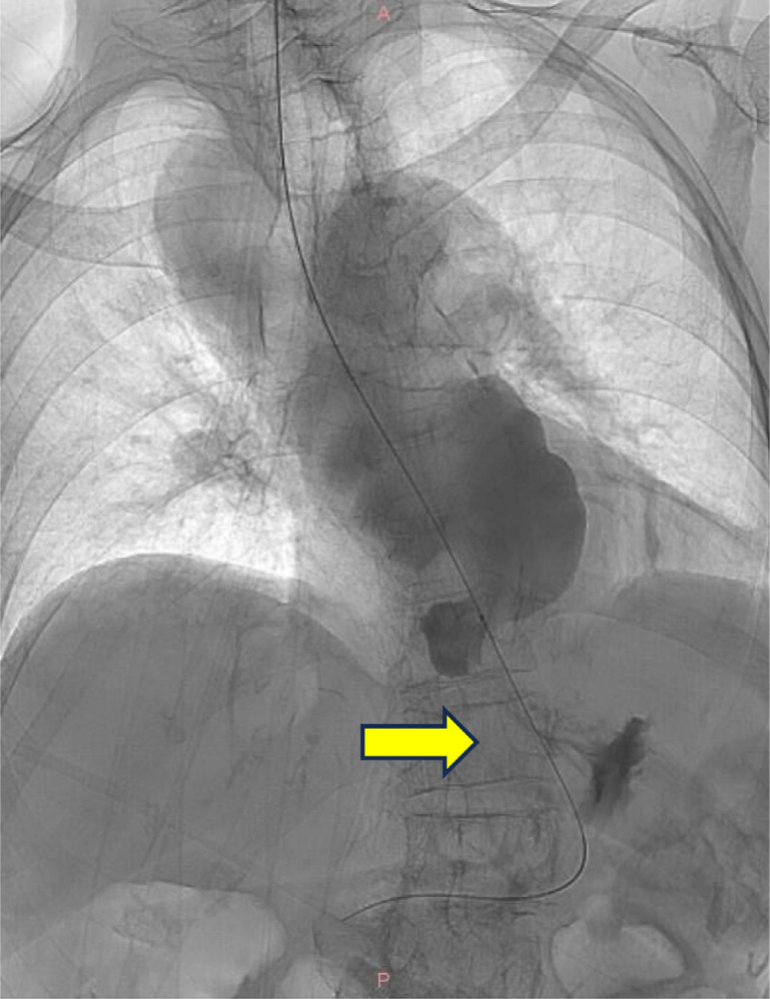
Fluoroscopy examination revealed a stricture in distal esophagus.

On examination, the patient appeared alert and cooperative but mildly undernourished. Her vital signs were stable, and her systemic examination was unremarkable, except for mild tenderness in the epigastric region. Laboratory investigations were within normal limits. A contrast-enhanced CT scan revealed a 4 cm stricture in the distal esophagus (Fig. 1). Upper gastrointestinal endoscopy showed that the scope could enter the lower esophageal sphincter (LES), which appeared narrowed and hyperemic, filled with food debris. The scope could not pass through the LES. Attempts to insert 14Fr and 16Fr nasogastric tubes were unsuccessful. Biopsies were taken from the LES mucosa. Histopathological evaluation revealed hyperplastic squamous epithelial cells with atypical nuclei and mild lymphocytic infiltration, but no evidence of malignancy. Fluoroscopy confirmed the presence and exact location of the distal esophageal stricture (Fig. 2).

**Fig. 3:**
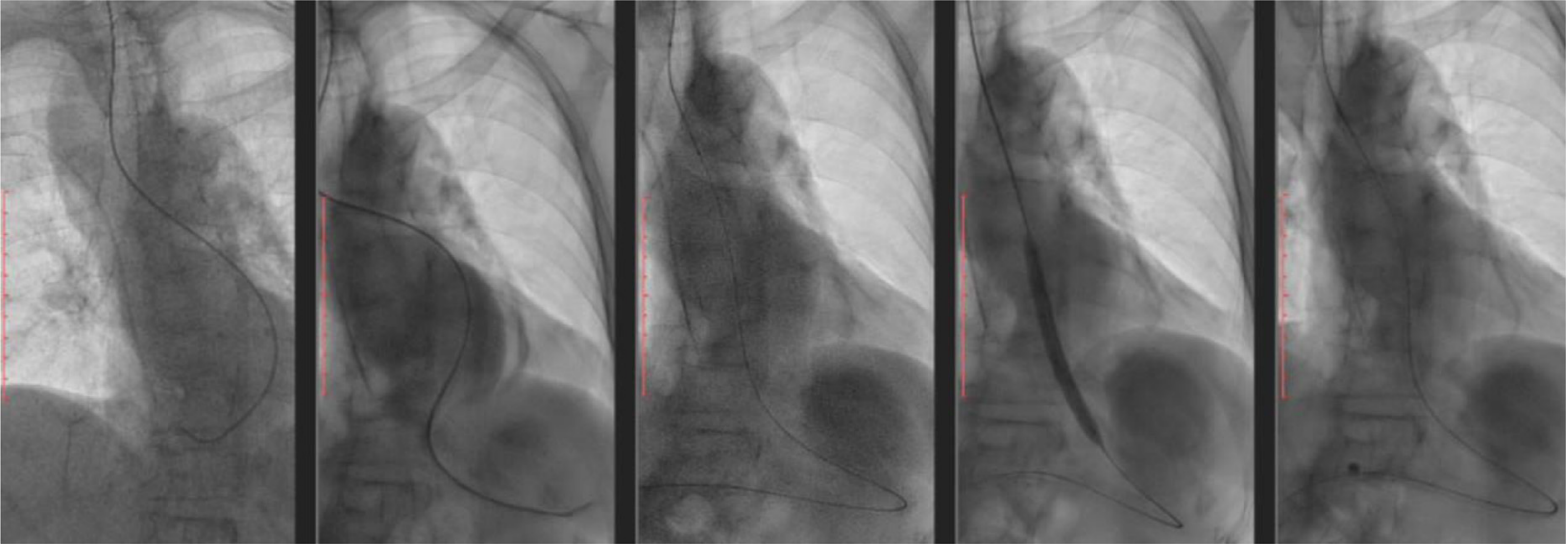
Balloon dilatation prior to esophageal stent placement on fluoroscopy.

**Fig. 4:**
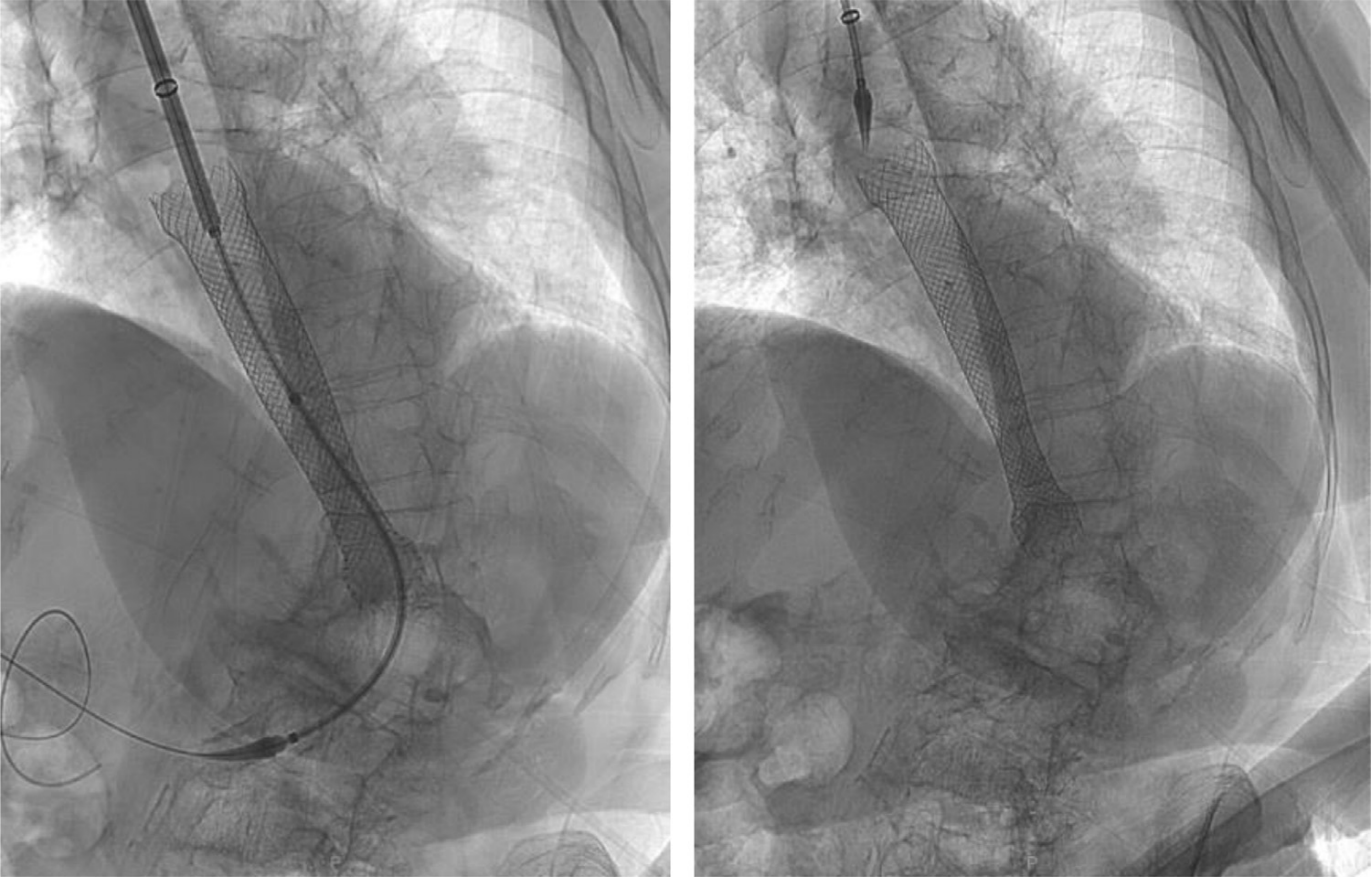
Placement of esophageal stent assisted with fluoroscopy.

**Fig. 5:**
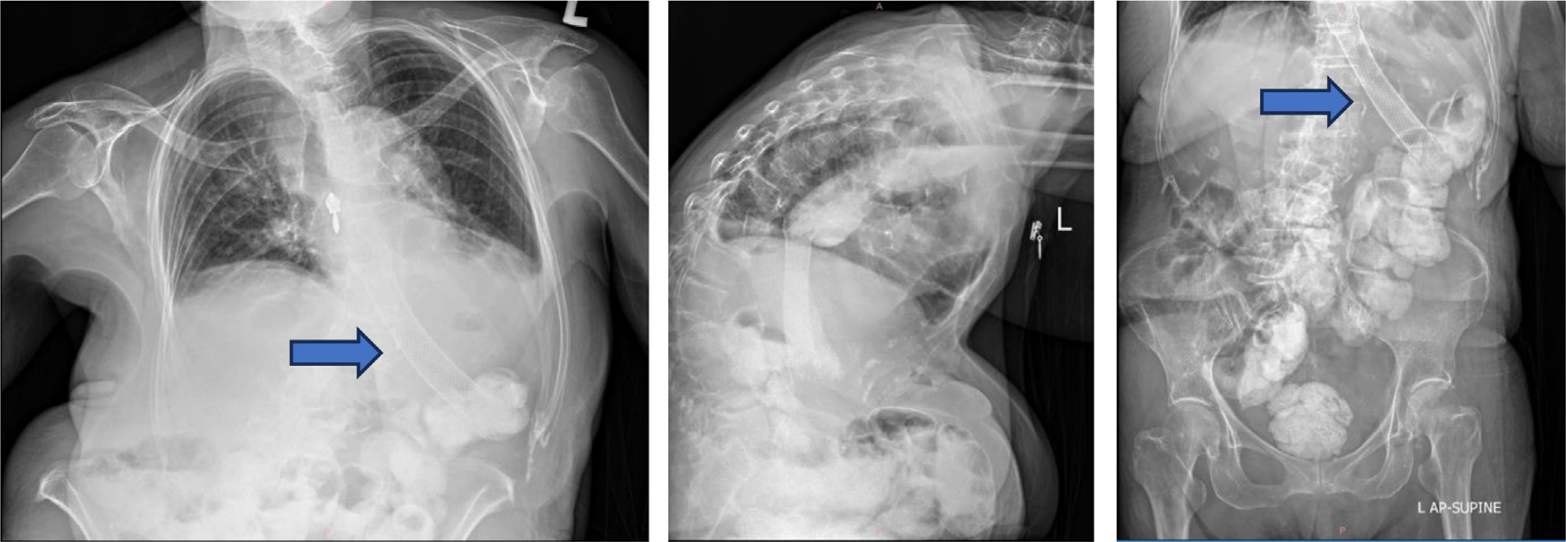
Esophageal stent shown on X-Ray imaging.

Given the severity and location of the stricture, esophageal stenting was planned. A WallFlex SEMS (Boston Scientific), equipped with an antireflux valve, was selected due to the patient’s history of GERD. Because of the extremely narrow lumen, the endoscope could not traverse the stricture, so fluoroscopy guidance was used. Prior to stenting, balloon dilatation was performed to allow passage of the stent delivery system (Fig. 3). The stent was successfully placed under fluoroscopic control (Fig. 4). Post-procedural imaging showed proper positioning of the stent (Fig. 5).

## Discussion

The esophagus is composed of multiple layers, including mucosa, submucosa, muscularis, and adventitia [7,8]. Pathologic narrowing of the esophagus can arise from various etiologies. In older adults, peptic strictures from GERD are the most common cause of benign esophageal strictures [1,9,10].

The progression from inflammation to fibrosis leads to luminal narrowing and symptomatic dysphagia. Chronic acid exposure, as seen in GERD, is a major contributor [1,11,12]. Malignant causes include adenocarcinoma (typically distal) and squamous cell carcinoma (more often proximal or mid-esophagus) [1,10].

In this patient, the slow progression of dysphagia and absence of malignant features in biopsy confirmed a benign etiology. Fluoroscopy played a crucial role in visualizing the lesion and guiding the stent placement, especially since endoscopic passage was not possible [13].

Dysphagia can be graded using the Ogilvie Dysphagia Score, where this patient scored a 3 (able to swallow liquids only) [14]. Nutritional assessment using the MUST tool was not performed but should be considered in similar cases [15].

Treatment options for esophageal stricture include endoscopic dilatation, stenting, and in rare cases, surgical intervention. Stenting is often preferred in cases with severe narrowing or failed dilatation. SEMS are particularly useful, though partially and fully covered SEMS are not FDA-approved for benign strictures [16,17]. SEMS with antireflux valves are beneficial for distal strictures to prevent worsening reflux symptoms [18,19]. In this case, an antireflux SEMS was chosen due to the patient’s preexisting GERD. Fluoroscopic placement allows precise deployment, and balloon dilation was needed in this case to allow passage of the stent system [18].

Complications such as stent migration, tumor ingrowth, and fistula formation are more common in the long term [20]. Regular follow-up is important to monitor for these outcomes.

## Conclusion

This case highlights the successful placement of an esophageal SEMS with antireflux features in a patient with a narrow distal esophageal stricture. Fluoroscopic guidance and balloon dilatation enabled the procedure despite the extremely narrow lumen. This report contributes to the growing body of literature on stenting in difficult esophageal stricture cases and may serve as a reference for similar future interventions.

## Patient consent

Written informed consent for publication of their case was obtained from our patient’s parent.
